# Comparisons of biochemical parameters and diabetic ketoacidosis severity in adult patients with type 1 and type 2 diabetes

**DOI:** 10.1186/s12902-021-00922-3

**Published:** 2022-01-06

**Authors:** Atchara Charoenpiriya, Laor Chailurkit, Boonsong Ongphiphadhanakul

**Affiliations:** 1 Endocrine and Metabolism Unit, Department of Medicine, Maharaj Nakhon Si Thammarat Hospital, Nakhon Si Thammarat, 80000 Thailand; 2 Division of Endocrinology and Metabolism, Department of Medicine, Faculty of Medicine, Ramathibodi Hospital, Mahidol University, Bangkok, 10400 Thailand

**Keywords:** Beta-hydroxybutyrate, Diabetic ketoacidosis, Type 1 diabetes, Type 2 diabetes

## Abstract

**Objective:**

The aim of this study was to determine the differences in biochemical parameters and diabetic ketoacidosis (DKA) severity in adult patients with type 1 and type 2 diabetes and utilization of serum BHB as a biomarker for DKA resolution was also evaluated.

**Materials and methods:**

This prospective observational study of type 1 or type 2 diabetes mellitus who were diagnosed with DKA between 01 October 2018 and 30 September 2020. The correlations between serum BHB, measured by the Ranbut assay, and pH, bicarbonate, and anion gap were examined.

**Results:**

A total of 99 diabetes patients were diagnosed with DKA (mean age 39.4 years, 63.4% female, 53.6% T2DM). while infection was the most common precipitating factor in T2DM (43.4%), non-compliance with treatment was the most common precipitating factor in T1DM (43.5%). T1DM patients had more severe DKA more hypokalemia during treatment. However, there was no significant difference in mortality between type1 and type2 diabetes. The initial laboratories evaluation of patients did not significant differ between type1 and type2 diabetes. Serum BHB during treatment of DKA was significantly correlated with changes in serum bicarbonate (*r* = − 0.64), serum anion gap (*r* = 0.84), and venous pH (*r* = − 0.6). The serum BHB levels corresponding to HCO_3_ levels for DKA severity were 4.5, 5.7, and 5.9 mmol/L in mild, moderate, and severe DKA, respectively. The serum BHB level of < 1 mmol/L had 73.7% sensitivity and 100% specificity to predict DKA resolution. Median time to resolution of DKA was 12 h with an optimized BHB cut-off value of < 1 mmol/L. There were no significant difference in time to resolution of DKA in the patients with type 1 and type 2 diabetes.

**Conclusions:**

There are no differences in DKA-related biochemical parameters between type 1 and type 2 diabetes patients. The present findings suggest that DKA should be assessed and treated similarly, regardless of its occurrence in type 1 or type 2 diabetes patients.

## Introduction

Diabetic ketoacidosis (DKA) is a life-threatening acute complication of diabetes mellitus characterized by hyperglycemia and ketoacidosis. It can occur in both type 1 diabetes and type 2 diabetes patients under stress conditions such as infection, surgery, and trauma or under administration of SGLT2 inhibitors. The mortality rate for patients with DKA is about 1% and the rate rises to 5% in elderly patients [1, 2]. Multiple studies of DKA have been carried out in Caucasian populations mainly with type1 diabetes. However, a significant proportion of DKA in Asian populations occurs in type 2 diabetes [3–6] and it is unclear if clinical courses of DKA of type 1 and type 2 diabetes in Asian population differ.

The severity of DKA is generally classified as mild, moderate, or severe according to the American Diabetes Association. However, variations in the criteria exist among different professional societies. It should be noted that the severity criteria for DKA were mainly derived in Western countries, and that it remains unclear whether the criteria are appropriate for Asian populations and whether the severity of DKA is associated with clinical characteristics, biochemical derangements, and outcomes in Asian populations. Furthermore, it is currently unknown whether the severity of DKA based on clinical and biochemical parameters differs in patients with type 1 and type 2 diabetes.

Laboratory findings in DKA consist of hyperglycemia (> 250 mg/dL), high anion gap metabolic acidosis, and detection of serum ketone or urine ketone bodies [7]. Three types of ketone bodies, acetoacetate, beta-hydroxybutyrate (BHB), and acetone, are produced in DKA. The ratio of BHB to acetoacetate in patients with DKA is increased by 10 times compared with the healthy population [8]. The nitroprusside test, which is widely used to assess ketosis, can provide a high false-negative rate in DKA because nitroprusside reacts with acetoacetate and acetone, but not with BHB [9]. Recently, the nitroprusside test has been replaced by direct measurement of serum BHB. The serum BHB cut-off point of 3 mmol/L in diagnosis of DKA provides 90–100% sensitivity and 86–100% specificity [10–15]. However, the cutoff point of BHB in resolution of DKA remains unclear. The purpose of the present study was to determine the clinical and biochemical features and severity of DKA in Thai adult patients with type 1 and type 2 diabetes and utilization of serum BHB as a biomarker for DKA resolution was also evaluated.

## Materials and methods

This prospective observational study recruited all 99 patients with type 1 or type 2 diabetes mellitus who were diagnosed with DKA between 01 October 2018 and 30 September 2020 at our hospital.

### Study subjects

Patients were assigned to the type 2 diabetes group if they were previously diagnosed and managed with diet and exercise alone or with oral hypoglycemic agents or with insulin but not on a regular basis. Patients with DKA whose medical records indicating prior diagnosis of type 1 diabetes or if serum C peptide was below 1 ng/mL and received regular insulin treatment at follow-up were regarded as type 1 diabetes. Patients with newly diagnosed diabetes were classified as type 1 diabetes if the level of C peptide below 1 ng/mL after resolution of DKA for 4 weeks.

The inclusion criteria were: age > 18 years, diagnosed with DKA and admitted to Maharaj Nakorn Si Thammarat Hospital, serum blood glucose ≥250 mg/dL and serum BHB ≥3 mmol/L, and venous blood pH < 7.3 or serum bicarbonate < 18 mmol/L [11]. We classified the severity of DKA at the time of diagnosis into mild, moderate, and severe according to the 2009 consensus statement of the American Diabetes Association [7] as follows: mild DKA, pH > 7.25 and HCO_3_ ≥ 15 mmol/L or anion gap > 10; moderate DKA, pH 7.0–7.24 and HCO_3_ 10–15 mmol/L or anion gap > 12; severe DKA, pH < 7.0 and HCO_3_ < 10 mmol/L or anion gap > 12. Serum BHB, venous pH, and electrolytes were followed up every 4 h until DKA was resolved. Resolution of DKA was defined as plasma glucose < 200 mg/dL plus any two of bicarbonate > 15 mmol/L, pH > 7.3, and anion gap < 12 mmol/L. Patients were excluded if they were unable to provide informed consent or had wide gap metabolic acidosis from other conditions (eGFR < 60 mL/min/1.73 m^2^), drug-induced acidosis, chronic alcohol drinking, pregnancy, and serum lactate > 18 mg/dL (2 mmol/L) [12].

### Biochemical measurements

Serum BHB was measured by an enzymatic method, the Ranbut assay, obtained from Randox Laboratories Limited (United Kingdom). The test was linear for serum BHB at 0.1–5.75 mmol/L and had a correlation coefficient of *r* = 0.9954. Serum glucose, blood urea nitrogen (BUN), creatinine, serum lactate, and serum electrolytes were measured using a Cobas 6000 analyzer series (c501 module).

### Ethical considerations

This study was approved by the medical ethics committee of Maharaj Nakhon Si Thammarat Hospital, and the written informed consent was obtained from all participants according to the guidelines of Declaration of Helsinki. All methods in this study were performed in accordance with the relevant guidelines and regulations.

### Statistical analysis

STATA® version 14 software was used for the statistical analyses. Descriptive statistics were measured by the patient number, percentage, or mean (min–max) as appropriate. Correlations between BHB and pH, bicarbonate, and anion gap were established by Pearson correlation analysis. Values of *p* < 0.05 were considered to indicate statistical significance. Serum BHB according to DKA severity was reported in median and interquartile range. Wilcoxon rank-sum test to assess the statistical difference in the median.

## Results

Between 01 October 2018 and 30 September 2020, 99 adult patients with diabetes mellitus were diagnosed with DKA at Maharaj Nakhon Si Thammarat Hospital, Thailand. The mean age was 39.4 years (range, 18–85 years). Most of the patients were female (63.4%). The proportions of DKA predominant in type2 diabetes (53.6%). While infections were the most common precipitating factor for DKA in T2DM (43.4%), non-compliance with treatment was the most common precipitating factor of DKA in T1DM (43.5%). Diabetes was first diagnosis in DKA episode was 17.2% (T1DM 17.4%, T2DM17%). Treatment diabetes before DKA episode mostly used insulin 58.6% (T1DM 82.6, T2DM 37.7%). More than half of the patients were considered to have severe DKA (T1DM 67.4%, T2DM 41.5), 27.3% had moderate DKA (T1DM19.6%, T2DM34%), and 19.2% had mild DKA (T1DM13%, T2DM24.5%). Serum glucose ranged from 250 to 1243 mg/dL, with a mean of 490 mg/dL. Level of glycated hemoglobin was 12.4% (range, 8.3–17.5%). Effective serum osmolarity was 291 mOsm/Kg (range, 252-360 mOsm/kg). All DKA patients with high effective serum osmolarity more than 320mOs/kg were T2DM (11.3%). All patients were acidotic and the mean serum BHB was 5.9 mmol/L (range, 3.0–10.6 mmol/L). The other details of baseline characteristics and laboratory data are shown in Table 1. Patients with type 2 diabetes were older, higher BMI, longer duration of diabetes, presented DKA combined with hyperosmolarity and had higher serum pH, lactate, and HCO_3_, while patients with type 1 diabetes more commonly had severe DKA and hypokalemia after treatment.

**Table 1:** Clinical characteristics of the study population (*N* = 99)

	All patients	Type 1 diabetes	Type 2 diabetes	*p*-value
Patients, *n* (%)	99 (100)	46 (46.4)	53 (53.6)	
Age (y), mean (min–max)	39.4 (18–85)	26.5 (18–47)	50.5 (20–85)	< 0.001
Male sex, *n* (%)	36 (36.6)	18 (39.1)	18 (33.9)	0.59
BMI (kg/m^2^)^a^, mean (min-max)	20 (14.2–46.8)	19.9 (14.2–27.7)	22.0(14.8–46.9)	0.002
Duration of diabetes (year)	6.3 (0–25)	4.8 (0–15.3)	7.5 (0–25)	0.02
Precipitating factor, *n* (%)				
Non-compliance with treatment	32(32.3)	20(43.5)	12(22.6)	0.118
Infections	37(37.3)	14(30.4)	23(43.4)	
Newly diagnosed	17(17.2)	8(17.4)	9(17.0)	
Other major stresses	7(7.1)	1(2.2)	6(11.3)	
No cause identified	6(6.1)	3(6.5)	3(5.7)	
Glucose-lowering drug *n* (%): some patients received more than one drug				
Metformin	46(46.5)	7(15.2)	39(73.6)	< 0.001
Sulfonylurea	31(31.3)	0(0)	31(58.5)	< 0.001
Thiazolidinedione	11(11.1)	2(4.4)	9(17)	0.05
Insulin	58(58.6)	38(82.6)	20(37.7)	< 0.001
Severity of DKA, *n* (%)				
Mild	19 (19.2)	6 (13.0)	13 (24.5)	< 0.05
Moderate	27 (27.3)	9 (19.6)	18 (34.0)	
Severe	53 (53.5)	31 (67.4)	22 (41.5)	
Initial laboratory findings, mean (min–max)				
Plasma glucose (mg/dL)	490 (250–1243)	486 (138–1088)	494 (241–1243)	0.83
Glycated hemoglobin (%)	12.4 (8.3–17.5)	12.3 (8.3–17.5)	12.4 (8.7–15.8)	0.83
Serum BUN (mg/dL)	19.9 (5–71)	19.3 (7–48)	20.5 (5–71)	0.60
Serum creatinine (mg/dL)	0.75 (0.27–1.46)	0.75 (0.27–1.41)	0.74 (0.35–1.46)	0.83
eGFR (CKD-EPI, mL/min/1.73m^2^)	107 (60–194)	119 (62–194)	98.5(60–144)	0.001
Effective serum osmolarity (mOsm/kg)^b^	291(252–360)	289(251–316)	293(252–360)	0.21
Effective serum osmolarity > 320 mOsm/kg^b^*n* (%)	6(6.1)	0(0)	6(11.3%)	0.02
Free T4 (ng/dL) (normal range = 0.93–1.71)	1.2(0.8–4.1)	1.2(0.8–4.1)	1.2(0.8–1.9)	0.27
TSH (mIU/L) (normal range = 0.27–4.2)	1.8(0.05–13.2)	1.9(0.05–13.2)	1.9(0.35–5.0)	0.91
Serum Na^+^ (mmol/L)	132 (115–158)	131 (115–146)	133 (117–158)	0.19
Corrected serum Na^+^(mmol/L)^c^	138 (120–169)	137(122–151)	139(120–169)	0.17
Serum K^+^ (mmol/L)	4.4 (2.4–7.5)	4.5 (2.4–7.5)	4.3 (2.4–6.6)	0.35
Serum Cl^−^ (mmol/L)	96 (73–127)	95 (74–113)	96 (73–127)	0.59
Serum HCO_3_^−^ (mmol/L)	9.6 (2.0–23.5)	8.4 (2.0–18.2)	10.7 (3.0–23.5)	0.02
Anion gap (mmol/L)^d^	26.6 (12.5–42.8)	27.4 (14.4–42.8)	25.9 (12.5–41.7)	0.24
pH	7.18 (6.68–7.42)	7.14 (6.70–7.41)	7.21 (6.68–7.42)	0.02
BHB (mmol/L)	5.9 (3.0–10.6)	6.0 (3.4–9.0)	5.8 (3.0–10.6)	0.55
Lactate (mg/dl)	11.7 (4.9–18.0)	10.7 (4.9–18.0)	12.6 (5.6–18.0)	0.01
Time to DKA resolution (h), mean (min–max)	20.4 (8–64)	22 (8–64)	19.1 (8–60)	0.17
Complications during DKA treatment, *n* (%), events	50 (50.5), 59	21 (45.6), 25	29 (54.7), 34	0.81
Hypoglycemia (blood sugar < 70 mg/dL)	4 (4.04)	1 (2.2)	3 (5.7)	0.77
Hypokalemia (K^+^ < 3.5 mmol/L)	44 (44.4)	21 (45.7)	23 (43.4)	0.05
Hypernatremia (Na^+^ > 145 mmol/L)	11 (11.1)	3 (6.5)	8 (15.1)	0.83
Mortality	0 (0)	0 (0)	0 (0)	0 (0)
Complications at time of DKA resolution, *n* (%)				
Hypokalemia (K^+^ < 3.5 mmol/L)	34 (34.3%)	17 (37.0%)	17 (32.1%)	0.26
Hyponatremia (Na^+^ < 135 mmol/L)	24 (24.2%)	12 (26.1%)	12 (22.6%)	0.16
Hypernatremia (Na^+^ > 145 mmol/L)	10 (10.1%)	2 (4.4%)	8 (15.1%)	0.13

^a^The body-mass index is weight in kilograms divided by the square of height in meters

^b^Effective serum osmolarity: 2[measured Na^+^(mmol/L)] + glucose(mg/dL)/18

^c^Corrected serum Na^+^(mmol/L): measured (Na+) + [1.6(glucose in mg/dL-100)/100]

^d^Anion gap: (Na^+^) - [(Cl^−^ + HCO_3_^−^]

**Fig. 1:**
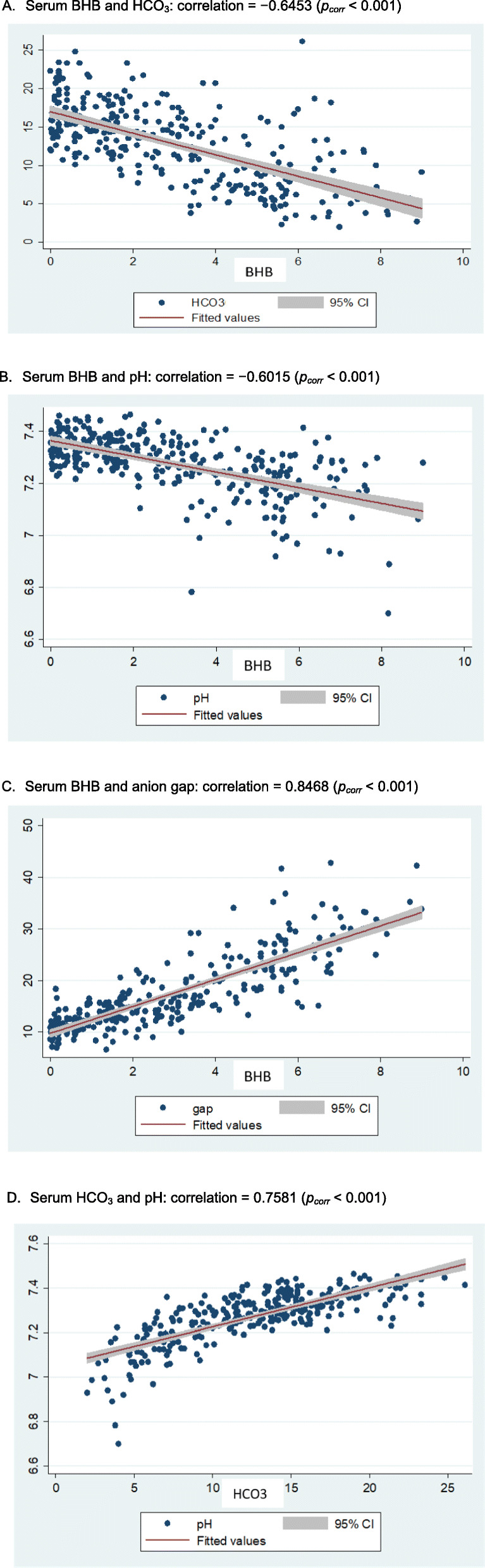
Correlations between serum BHB and bicarbonate (**A**), serum BHB and pH (**B**), serum BHB and anion gap (**C**), and serum bicarbonate and pH (**D**) in type 1 diabetes patients with DKA during treatment. **A** Serum BHB and HCO_3_: correlation = − 0.6453 (*p*_*corr*_ < 0.001), **B** Serum BHB and pH: correlation = − 0.6015 (*p*_*corr*_ < 0.001). **C** Serum BHB and anion gap: correlation = 0.8468 (*p*_*corr*_ < 0.001). **D** Serum HCO_3_ and pH: correlation = 0.7581 (*p*_*corr*_ < 0.001)

**Fig. 2:**
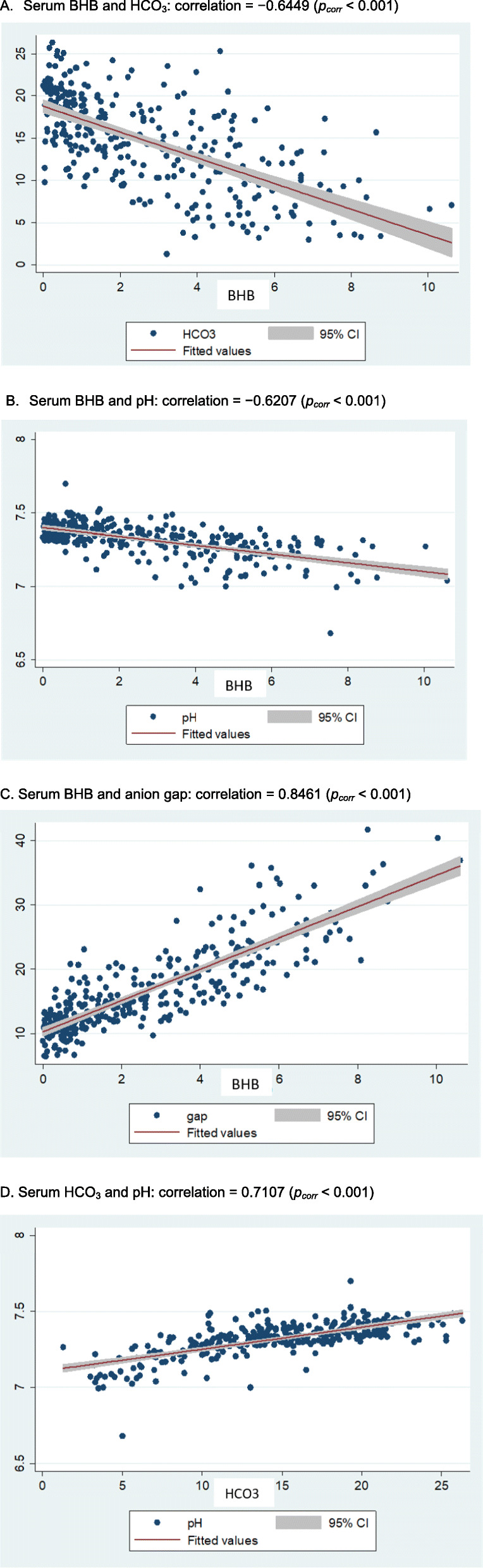
Correlations between serum BHB and bicarbonate (**A**), serum BHB and pH (**B**), serum BHB and anion gap (**C**), and serum bicarbonate and pH (**D**) in type 2 diabetes patients with DKA during treatment. **A** Serum BHB and HCO_3_: correlation = − 0.6449 (*p*_*corr*_ < 0.001). **B** Serum BHB and pH: correlation = − 0.6207 (*p*_*corr*_ < 0.001). **C** Serum BHB and anion gap: correlation = 0.8461 (*p*_*corr*_ < 0.001). **D** Serum HCO_3_ and pH: correlation = 0.7107 (*p*_*corr*_ < 0.001)

The relationships among biochemical parameters during treatment of DKA were investigated. For type 1 diabetes patients (Fig. 1), there were negative correlations between serum BHB and HCO_3_^−^(− 0.6453), serum BHB and pH (− 0.6015), and positive correlations between serum BHB and anion gap (0.8468), serum HCO_3_^−^ and pH (0.7581). Likewise, in type 2 diabetes patients (Fig. 2), there were negative correlations between serum BHB and HCO_3_^−^ (− 0.6449), serum BHB and pH (− 0.6207) and positive correlations between serum BHB and anion gap (0.8461), serum HCO_3_^−^ and pH (0.7107). All of the corresponding correlations appeared comparable between the type 1 and type 2 diabetes patients.

Regarding the severity of DKA, the median serum BHB levels increased with increasing DKA severity. In all patients, the median serum BHB levels were 4.5 mmol/L (IQR, 3.9–5.8) for mild DKA, 5.7 mmol/L (IQR, 4.7–6.7) for moderate DKA, and 5.9 mmol/L (IQR, 5.3–7.4) for severe DKA. In each severity category, there was no significant difference in the serum BHB levels between the patients with type 1 or type 2 diabetes (Table 2).

**Table 2:** Serum BHB levels according to type of DKA and grading severity of DKA

	Mild DKA^a^	Moderate DKA^a^	Severe DKA^a^
Serum BHB in type 1 diabetes patients (mmol/L), median (IQR)	4.7 (4.0–5.9)	5.5 (4.9–7.0)	5.7 (5.4–7.0)
Serum BHB in type 2 diabetes patients (mmol/L), median (IQR)	4.5 (3.9–5.0)	5.7 (4.6–6.7)	6.3 (5.2–7.7)
serum BHB in all diabetes patients (mmol/L), median (IQR)	4.5 (3.9–5.8)	5.7 (4.7–6.7)	5.9 (5.3–7.4)

^a^Grading severity for DKA was taken from to the 2009 consensus statement of the American Diabetes Association

When DKA has resolved, BHB level is only 0.75 mmol/L (0.6–0.9, *p* < 0.01). The BHB level corresponding to the HCO_3_ criteria was 1.05 mmol/L (0.22–1.8, *p* = 0.013). However, BHB levels was not correlate to blood pH. Sensitivity and specificity of BHB cut-of values for the determination of the resolution of DKA are shown in Table 3.

**Table 3:** Resolution of DKA (from ADA criteria) and the correlation of serum BHB with pH and [HCO_3_^−^]

		Sensitivity	Specificity
Blood sugar	< 200 mg/dL		
Arterial pH^a^	> 7.3		
Serum bicarbonate (mmol/L) or [HCO_3_^−^]^a^	> 15		
Anion gap (mmol/L) ^a^	< 12		
BHB (mmol/L)	0.75 (0.62–0.90), *p* < 0.01	58.6%	100%
BHB corresponding to [HCO_3_^−^] (mmol/L)	1.05 (0.22–1.87), *p* = 0.013	73.7%	100%
BHB corresponding to pH (mmol/L)	4.56 (−11.9–21.0), *p* = 0.58	100%	76.7%

^a^The resolution criteria for DKA from ADA2009

Median time to DKA resolution, as defined by BHB < 1 mmol/L, serum HCO_3_^−^ > 15 mmol/L and anion gap < 12 mmol/L was 12 h. Not unexpectedly, pH > 7.3 appeared to be the main determinant of shorter resolution time. In subject with pH > 7.3, median time to resolution of DKA in type 2 diabetes was shorter than those with type 1 diabetes patients (Table 4).

**Table 4:** Median hour to DKA resolution (IQR) by diabetes type

Variables	Median time to DKA resolution in hour (IQR: Q1 to Q3)			*p*-value (Wilcoxon rank-sum test)
	All patients	Type 1 diabetes	Type 2 diabetes	
BHB < 1 mmol/L	12 (8 to 16)	12 (8 to 20)	12 (8 to 16)	0.14
BHB < 1 mmol/L and pH > 7.3	10 (6 to16)	12 (8 to 18)	8 (6 to 12)	0.06
BHB < 1 mmol/L and serum HCO_3_^−^ > 15 mmol/L	11 (8 to16)	12 (8 to 20)	8 (6 to 14)	0.42
BHB < 1 mmol/L and anion gap < 12 mmol/L	12 (8 to 16)	14 (8 to 20)	12 (8 to 16)	0.25
pH > 7.3	8 (4 to 12)	8 (4 to 16)	4 (4 to 8)	0.04
pH > 7.3 and serum HCO_3_^−^ > 15 mmol/L	8 (4 to 14)	12 (6 to 16)	6 (4 to 12)	0.05
pH > 7.3 and anion gap < 12 mmol/L	10 (6 to14)	12 (8 to 16)	8 (6 to 12)	0.01
Serum HCO_3_^−^ > 15 mmol/L	12 (4 to 16)	12 (8 to 20)	8 (4 to 16)	0.06
Serum HCO_3_^−^ > 15 mmol/L and anion gap < 12 mmol/L	12 (8 to 16)	14 (8 to 18)	10 (6 to14)	0.26
Anion gap < 12 mmol/L	12 (8 to 16)	12 (8 to 20)	12 (8 to 16)	0.27

## Discussion

DKA can occur in both type 1 and type 2 diabetes patients, although the incidence was reported to be much higher in patients with type 1 diabetes in a Caucasian population [3, 4, 16]. In the present study, we found that the DKA occurred frequently in patients with type 2 diabetes, which is similar to previous studies in Asian populations [5, 6]. This may be partly due to the very low prevalence type 1 with DKA in Thailand [17] as well as other countries in the tropics [18, 19] although ethnic or genetic variations in Asian patients with type 2 diabetes which might make them more prone to develop DKA cannot be ruled out [20]. In western countries, during the last two decades, hospitalizations for DKA at least in England have increased for both adult patients with type 1 diabetes and adult patients with type 2 diabetes [21].

In the present study, the initial laboratory evaluation of type 1 and type 2 patients including plasma glucose, glycated hemoglobin, blood urea nitrogen, creatinine, electrolyte, osmolarity, thyroid function test and serum BHB did not significant differ. Our results showed that precipitating factors for DKA are different in type 1 and type 2 diabetes. In our type 1 patients, discontinuation of treatments was the most common cause for the development of DKA while infection was a major precipitating factor in type 2 diabetes. With regard to mortality although the patients with type1 diabetes with DKA in our study had higher severity of the disease and more hypokalemia during treatment, there was no significant difference in mortality between DKA patients who had type 1 or type 2 diabetes, probably because of our study populations were younger than previous studies [3, 4] and a less severe disease because of the exclusion of other causes of acidosis To our knowledge, no investigations have examined the influence of type of diabetes on the relationship between DKA severity using common assessment methods and DKA outcomes. The present findings suggest that DKA should be assessed and treated similarly, regardless of its occurrence in type 1 or type 2 diabetes patients.

The findings of the present study demonstrate that the changes in serum BHB during treatment of DKA are significantly correlated with the changes in serum bicarbonate, serum anion gap, and venous pH, and are consistent with the findings from a number of previous studies. A retrospective study reported a negative correlation between serum BHB and bicarbonate (*r* = − 0.64; *p* < 0.001) [13]. Similarly, a prospective study demonstrated a negative correlation between serum BHB and bicarbonate (*r* = − 0.69) as well as a positive correlation between serum BHB and anion gap (*r* = 0.75) [14]. Negative correlations between serum BHB and pH (*r* = − 0.67) and serum BHB and bicarbonate (*r* = − 0.7) have also been reported [15]. With regard to capillary BHB, it was analyzed in a study on type 1 diabetes patients who developed DKA [22]. The study found correlations between capillary BHB and pH (*r* = − 0.56419; *p* < 0.0001) and capillary BHB and bicarbonate (*r* = − 0.24139; *p* = 0.0161). In the present study, the relationships between serum BHB and relevant parameters for monitoring of DKA did not differ significantly between type 1 and type 2 diabetes patients. These findings suggest that serum BHB can be used as a parameter for monitoring clinical symptoms in patients during DKA regardless of the type of diabetes. It should be noted that serum BHB provides better accuracy for assessment of DKA than capillary blood BHB [22], and the utilization of capillary blood BHB in this regard is unclear.

In the present study, we found upward trends for serum BHB with increasing severity of DKA that were similar between type 1 and type 2 diabetes patients. Our trends for serum BHB are consistent with findings for patients with type 2 diabetes [15] and children and adults with uncontrolled diabetes [23]. The former study found that the serum BHB levels corresponding to serum bicarbonate levels of 18, 15, and 10 mmol/L were 3.0 mmol/L for mild DKA, 4.7 mmol/L for moderate DKA, and 7.5 mmol/L for severe DKA. The latter study found the same trends that the serum BHB levels corresponding to serum bicarbonate levels of 18, 15, and 10 mmol/L were 3.8, 5.1, and 8.9 mmol/L, respectively. A study from the Joint British Diabetes Society in 2011 found that presence of capillary BHB > 6 mmol/L indicated severe DKA [11]. Based on our data and the results from previous studies, we suggest that serum BHB levels can be categorized into < 5 mmol/L for mild DKA, 5–6 mmol/L for moderate DKA, and > 6 mmol/L for severe DKA, and should be similar in patients with type 1 and type 2 diabetes.

In the present study, BHB levels were not only associated with the severity of DKA but also related to the resolution of DKA as assessed by other biochemical parameters. Previous studies [11, 24, 25] have suggested that a BHB cut-off value of 0.6–1.5 mmol/L provided as definitions for DKA resolution. We found that BHB level < 1 mmol/L and any two of the following parameters including bicarbonate> 15 mmol/L, pH > 7.3 and anion gap< 12, BHB has acceptable sensitivity and good specificity for the resolution of DKA. The upward trend of BHB and serum HCO_3_^−^ were from the volume depletion on admission [26]. In our study, median time to resolution of DKA defines by pH > 7.3 and a composite of pH > 7.3 and serum HCO_3_^−^ > 15 mmol/L was the shortest which may be due to the fact that pH is more sensitive to rapid changes in respiratory status. It is of note that the median time to DKA resolution in type2 diabetes was significantly shorter than in type 1 diabetes patients, which may be that in our study, patients with type 1 diabetes had more severe DKA. Clinicians must use clinical judgment when interpreting acid-base disorder during DKA treatment. We have shown that resolution of DKA as defined by BHB < 1 mmol/L can be reasonably predicted by a composite of serum HCO_3_^−^ > 15 mmol/L and anion gap < 12 mmol/L according to the ADA guidelines [7]. Therefore, serum BHB may be widely used as a parameter for monitoring and resolution clinical symptoms of patients during DKA. It is also useful in differentiating DKA from other conditions presenting with wide gap metabolic acidosis i.e. sepsis and renal failure. More reliable laboratory measurements may assist in reducing time to recovery and costs of hospital stay.

Our study is limited by exclusively using the Ranbut assay for BHB testing and comparison with other generally applied tests was not performed. Moreover, a single BHB cutoff value was used in both type 1 and type 2 diabetes as it is currently unclear if the threshold would be different in DKA patients with type 1 and type 2 diabetes.

## Data Availability

The datasets used and analyzed during the current study are available from the corresponding author on reasonable request.

## Abbreviations

DKA: Diabetic ketoacidosis
BHB: Beta-hydroxybutyrate
HCO_3_: Bicarbonate
SGLT2: Sodium glucose co-transporter type 2 (SGLT2)

## References

[1] Graves EJ, Gillum BS. Detailed Diagnoses and Procedures, National Hospital Discharge Survey, 1995. Vital and Health Statistics. Series 13, Data from the National Health Survey, no. 130 (November). 1997;1–146. 9429338

[2] Malone ML, Gennis V, Goodwin JS (1992). Characteristics of diabetic ketoacidosis in older versus younger adults. J Am Geriatr Soc.

[3] Barski L (2013). Comparison of diabetic ketoacidosis in patients with type-1 and type-2 diabetes mellitus. Am J Med Sci.

[4] Newton CA, Raskin P (2004). Diabetic ketoacidosis in type 1 and type 2 diabetes mellitus: clinical and biochemical differences. Arch Intern Med.

[5] Tan H, Zhou Y, Yu Y (2012). Characteristics of diabetic ketoacidosis in Chinese adults and adolescents -- a teaching hospital-based analysis. Diabetes Res Clin Pract.

[6] Thewjitcharoen Y (2019). Clinical characteristics and outcomes of care in adult patients with diabetic ketoacidosis: a retrospective study from a tertiary diabetes center in Thailand. J Clin Transl Endocrinol.

[7] Kitabchi AE (2009). Hyperglycemic crises in adult patients with diabetes. Diabetes Care.

[8] Laffel L (1999). Ketone bodies: a review of physiology, pathophysiology and application of monitoring to diabetes. Diabetes Metab Res Rev.

[9] Harris S (2005). Near patient blood ketone measurements and their utility in predicting diabetic ketoacidosis. Diabet Med.

[10] Tantiwong P (2005). Capillary blood beta-hydroxybutyrate measurement by reagent strip in diagnosing diabetic ketoacidosis. Clin Lab Sci.

[11] Savage MW (2011). Joint British Diabetes Societies guideline for the management of diabetic ketoacidosis. Diabet Med.

[12] Levey AS (2009). A new equation to estimate glomerular filtration rate. Ann Intern Med.

[13] Sheikh-Ali M (2008). Can serum beta-hydroxybutyrate be used to diagnose diabetic ketoacidosis?. Diabetes Care.

[14] Fulop M (1999). Serum beta-hydroxybutyrate measurement in patients with uncontrolled diabetes mellitus. Arch Intern Med.

[15] Ke P (2014). Establishment of blood beta-hydroxybutyrate threshold for diagnosis of type 2 diabetes ketoacidosis. Nan Fang Yi Ke Da Xue Xue Bao.

[16] Wang ZH, Kihl-Selstam E, Eriksson JW (2008). Ketoacidosis occurs in both type 1 and type 2 diabetes--a population-based study from northern Sweden. Diabet Med.

[17] Reutrakul S, Deerochanawong C (2016). Diabetes in Thailand: status and policy. Curr Diab Rep.

[18] Mobasseri M (2020). Prevalence and incidence of type 1 diabetes in the world: a systematic review and meta-analysis. Health Promot Perspect.

[19] Pinto ME, Villena JE, Villena AE (2008). Diabetic ketoacidosis in Peruvian patients with type 2 diabetes mellitus. Endocr Pract.

[20] Balasubramanyam A (1999). New profiles of diabetic ketoacidosis: type 1 vs type 2 diabetes and the effect of ethnicity. Arch Intern Med.

[21] Zhong VW, Juhaeri J, Mayer-Davis EJ (2018). Trends in hospital admission for diabetic ketoacidosis in adults with type 1 and type 2 diabetes in England, 1998-2013: a retrospective cohort study. Diabetes Care.

[22] Rodriguez-Merchan B (2011). Capillary beta-hydroxybutyrate determination for monitoring diabetic ketoacidosis. Endocrinol Nutr.

[23] Voulgari C, Tentolouris N (2010). The performance of a glucose-ketone meter in the diagnosis of diabetic ketoacidosis in patients with type 2 diabetes in the emergency room. Diabetes Technol Ther.

[24] Ham MR, Okada P, White PC (2004). Bedside ketone determination in diabetic children with hyperglycemia and ketosis in the acute care setting. Pediatr Diabetes.

[25] Wolfsdorf JI (2014). ISPAD clinical practice consensus guidelines 2014. Diabetic ketoacidosis and hyperglycemic hyperosmolar state. Pediatr Diabetes.

[26] Adrogue HJ (1982). Plasma acid-base patterns in diabetic ketoacidosis. N Engl J Med.

